# Draft genomes of three nitroguanidine-degrading bacteria: *Pseudomonas extremaustralis* NQ5*, Arthrobacter* strain NQ4, and *Arthrobacter* strain NQ7

**DOI:** 10.1128/MRA.00467-23

**Published:** 2023-07-21

**Authors:** Jinha Kim, Mark E. Fuller, Paul B. Hatzinger, Kung-Hui Chu

**Affiliations:** 1 Zachry Department of Civil and Environmental Engineering, Texas A&M University, College Station, Texas, USA; 2 Aptim Federal Services, Lawrenceville, New Jersey, USA; Indiana University, Bloomington, Bloomington, Indiana, USA

**Keywords:** biodegradation, insensitive munitions, nitroguanidine

## Abstract

We report the draft genome sequences of *Pseudomonas extremaustralis* NQ5, *Arthrobacter* strain NQ4, and *Arthrobacter* strain NQ7 isolated from a laboratory-scale membrane bioreactor, soils from San Antonio, TX, USA and sediments from Galveston Bay, TX, USA, respectively. These bacteria degrade the explosive compound nitroguanidine, which is present in some insensitive munitions.

## ANNOUNCEMENT

**Fig 1:**
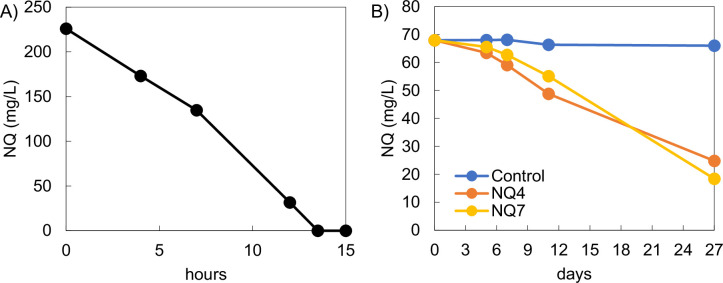
Time course of NQ degradation by (A) strain NQ5 and (B) strains NQ4 and NQ7, when NQ was supplied as sole nitrogen source and glucose was supplied as sole carbon source.

Nitroguanidine (NQ) is an explosive compound used in a variety of insensitive munitions developed to resist impact, friction, and heat (1). NQ is also used in the synthesis of some organic compounds, such as herbicides, and documented to cause acute and/or chronic toxicity in mice, aquatic organisms, and plants (2 - 4). *Pseudomonas extremaustralis* NQ5 was isolated from a laboratory-scale membrane bioreactor treating various explosive compounds, including NQ. *Arthrobacter* strain NQ4 was isolated from soil collected from a per- and polyfluoroalkyl substances-contaminated site in San Antonio, TX, USA. *Arthrobacter* strain NQ7 was isolated from sediments collected from Galveston Bay, TX, USA. Sample matrices were suspended and enriched in nitrogen-free mineral salts medium (N-free MSM) containing 2 mM NQ as sole nitrogen source and 11 mM glucose as sole carbon source for 3 weeks. The strains were isolated from N-free MSM agar plates with the same amendments by picking individual colonies. The colonies grew within a day (NQ5) or more than a week (NQ4 and NQ7) in N-free MSM with NQ and glucose as sole nitrogen and carbon sources, respectively (Fig. 1). NQ concentrations were analyzed by high-performance liquid chromatography using the method in Fuller et al. (5). Genomic DNAs (gDNA) were extracted using the FastDNA SPIN kit for soil (MP biomedical, Irvine, CA, USA) using the provided method. 16S rRNA genes were amplified using the 24F/1532R primer set. Sanger sequencing was conducted on both ends of the amplicons by Eton Biosciences (San Diego, CA, USA), and the trimmed paired-end reads were merged through VSearch (5). Through BLAST, NQ5, NQ4, and NQ7 were identified as *Pseudomonas extremaustralis* strain BF11 (MT441542.1), *Arthrobacter* strain AFS039412 (OP986498.1), and *Arthrobacter* strain SE3A52 (OQ121141.1), respectively, with 100% identity.

The same gDNAs used for 16S rRNA gene sequencing were quantified using a Qubit High Sensitivity (HS) Assay Kit on the Qubit Fluorometer 4.0 (Invitrogen, Waltham, MA, USA). The gDNA integrities were checked with the Genomic DNA ScreenTape Assay Kit (Agilent, Santa Clara, CA, USA) on a 4200 TapeStation (Agilent). Libraries were prepared with the Illumina DNA Prep Kit and IDT for Illumina UD indexes (Plate A) (Illumina, San Diego, CA, USA). Post-library quantification was conducted with the HS Assay Kit. Quality was checked with the D1000 ScreenTape Assay Kit (Agilent) on the 4200 TapeStation. Sequencing was performed through the MiSeq platform (Illumina) with the paired-end 2 × 250 bp strategy. Reads were trimmed, adapter removed, and quality controlled by FastQC within Trim Galore version 0.6.7 (6). The genomes were *de novo* assembled using SPAdes version 3.15.3 (7) on the Grace computing cluster at Texas A&M University. Annotated genomes of *Pseudomonas* sp. 02C 26 (CP025262.1), *Arthrobacter crystallopoietes* strain DSM 20117 (CP018863.1), and *Arthrobacter phenanthrenivorans* strain SWC37 (JWTB00000000) were used as references to identify contaminants within the NQ5, NQ4, and NQ7 assemblies that ran through the RASTtk-based custom annotation pipeline at BV-BRC (8, 9) (https://www.bv-brc.org/app/Annotation). Contaminants were removed using the BV-BRC Metagenomic Binning Tool (https://www.bv-brc.org/app/MetagenomicBinning). The filtered contigs were submitted and annotated through NCBI PGAP version 4.6 (10). Genome coverages were calculated with samtools version 1.16.1 (11) after mapping paired-end reads to the assemblies using BWA-MEM2 version 2.2.1 (12). Genomic features are provided in Table 1.

**TABLE 1:** Genomic features of strains NQ5, NQ4, and NQ7

Features	NQ5		NQ4		NQ7	
GenBank accession no.	JARBJR010000000		JARETC010000000		JARFXU020000000	
Assembly accession no.	GCA_029581615.1		GCA_029077345.1		GCA_029581595.2	
SRA^*a*^ accession no.	SRR23696550		SRR23696941		SRR23692575	
No. of paired-end reads	489,473		511,004		467,527	
No. of contigs	95		37		78	
Assembly length (bp)	6,533,326		4,483,921		4,874,196	
Genome coverage (×)	33		50		40	
Contig *N*_50_ (bp)	205,006		298,154		145,272	
Contig *L*_50_ (bp)	13		5		9	
GC content (%)	60.5		66.5		66.5	
No. of CDS^*b*^	6,038		4,074		4,560	
No. of CDS with proteins	5,908		4,052		4,522	
No. of complete rRNAs (5S, 16S, 23S)	2, 1, 1		2, 1, 1		3, 1, 0	
No. of predicted tRNAs	59		51		53	

^*a*^ Sequence Read Archive (SRA).

^*b*^ Coding sequences (CDS).

## Data Availability

Whole Genome Shotgun projects have been deposited in DDBJ/ENA/GenBank under accession numbers JARBJR000000000 (NQ5), JARETC000000000 (NQ4), and JARFXU000000000 (NQ7). The versions described in this paper are versions JARBJR010000000 (NQ5), JARETC010000000 (NQ4), and JARFXU020000000 (NQ7). BioProject accession numbers are PRJNA934168 (NQ5), PRJNA934170 (NQ4), and PRJNA934176 (NQ7). BioSample accession numbers are SAMN33268489 (NQ5), SAMN33268523 (NQ4), and SAMN33268549 (NQ7). The Sequence Read Archive (SRA) accession numbers are SRR23696550 (NQ5), SRR23696941 (NQ4), and SRR23692575 (NQ7).
